# Effects of Combined Interventions with Aerobic Physical Exercise and Cognitive Training on Cognitive Function in Stroke Patients: A Systematic Review

**DOI:** 10.3390/brainsci11040473

**Published:** 2021-04-08

**Authors:** Laura Amorós-Aguilar, Erica Rodríguez-Quiroga, Sara Sánchez-Santolaya, Margalida Coll-Andreu

**Affiliations:** Department of Psychobiology and Methodology of Health Sciences and Institute of Neurosciences, Universitat Autònoma de Barcelona, 08193 Bellaterra, Spain; Laura.Amoros@uab.cat (L.A.-A.); Erica.RodriguezQ@e-campus.uab.cat (E.R.-Q.); Sara.SanchezS@e-campus.uab.cat (S.S.-S.)

**Keywords:** stroke, aerobic physical exercise, cognitive rehabilitation, combined interventions, cognitive function

## Abstract

(1) Background: Stroke is a major cause of permanent disability in multiple functions, including the cognitive domain. Since both cognitive training and aerobic physical exercise may exert positive effects on cognition after stroke, one may expect synergistic benefits when combining both interventions. (2) Methods: We carried out a systematic search of studies testing, in adult stroke patients, whether structured aerobic exercise combined with cognitive training led to higher cognitive benefits than either of these interventions when applied singly, or than interventions not including aerobic exercise or cognitive training. (3) Results: Five fair-quality randomized controlled trials fulfilled the search criteria. Exercise intensity was moderate-vigorous and cognitive training was mainly computer-based. The studies were heterogeneous regarding the cognitive tests used, and for this reason, a meta-analysis was not performed. Only three studies included follow-up assessment. The combined intervention was associated with pre-post improvement in at least one cognitive test in all the studies, and with higher positive effects compared to other conditions (although statistical significance was not always reached) in four studies. (4) Conclusions: Further trials including a long-term follow-up and comprehensive neuropsychological testing should be undertaken to determine whether combined aerobic exercise and cognitive training leads to additive cognitive benefits after stroke.

## 1. Introduction

The global incidence of stroke is around 200 cases per 10,000 inhabitants, although estimates show regional differences and variations between developed and developing countries [1]. Stroke is the main cause of permanent disability and the second most common cause of dementia (the first being Alzheimer’s disease). Most risk factors are associated with aging (e.g., cardiovascular disorders, diabetes, and metabolic syndrome). It is not surprising, therefore, that the prevalence of stroke is higher in older people, but stroke can be suffered at any age, and in recent years, its prevalence has increased in younger adults [2,3].

Approximately 90% of stroke survivors suffer from chronic sequelae, and around 30% are unable to perform daily activities independently [4]. The most common sequelae occur in the motor, sensory, cognitive, and emotional domains [5,6]. Motor sequelae typically receive the most attention in rehabilitation settings, given their detrimental effects on autonomous living. However, the prevalence of chronic cognitive sequelae is also high. Some estimates suggest that around 32% of patients suffer cognitive deficit 3 years after a stroke [7], but the prevalence reported in several studies is much higher. For example, one study [8] found that 83% of patients exhibited impairment in at least one cognitive domain, while 50% of the patients in their sample were impaired in more than one domain. In addition, the risk of suffering from mild cognitive impairment and dementia after a stroke is significantly elevated compared to the general population [5,9]. In any case, improving the cognitive status of stroke patients can very positively impact their emotional state and quality of life [10]; it can contribute to improving their family and social relationships and may even facilitate their reincorporation to work and reduce the risk of dementia. Furthermore, there is a high degree of interdependence between cognitive and motor processes [11], so improved cognitive performance can contribute to the recovery of motor abilities, and vice versa [12].

Many cognitive rehabilitation strategies, usually focused on the specific domains that are impaired in each patient, are employed to treat cognitive disorders in stroke patients, principally by neuropsychologists and speech therapists. Some studies have revealed a number of the neural mechanisms involved in the efficacy of these interventions [13,14].

In addition, in recent years, evidence from animal research and human studies has accumulated indicating a potential role of aerobic physical exercise (PE) as another strategy capable of reducing cognitive impairment after stroke. Animal research has substantially contributed to revealing some of the varied neural mechanisms linked to the benefits of aerobic PE on cognitive recovery after a stroke, including increased synaptic plasticity, dendritic arborization, the proliferation and survival of adult-born neurons (neurogenesis), the reorganization of neural circuits, compensation mechanisms in surviving brain areas, angiogenesis, and decreased secondary injury and neuron loss [11,15,16,17]. Human studies in stroke patients give support, in general terms, to the effectiveness of PE as a strategy for cognitive recovery, although there are many discrepancies and unanswered questions regarding the optimal parameters of the exercise regime (intensity, frequency, duration of the intervention, initiation time after injury, etc.) [18,19,20,21]. Some of these studies have also given support to the neural benefits of physical exercise and physical activity [11,15], including reduced white matter damage [22], enhanced interhemispheric connectivity of the dorsal attention network [23], and greater blood flow in the medial temporal lobe [24].

If both aerobic PE exercise and cognitive training exert positive cognitive and neural effects when applied singly, combining both kinds of interventions may be expected to yield to summative effects, in line with the findings in people with mild cognitive impairment [25,26,27]. However, to our knowledge, the efficacy of this combined strategy in stroke patients has not been the scope of previous reviews.

In view of these considerations, the aims of this systematic review were (1) to determine whether the combination of supervised aerobic PE plus structured cognitive training results in improved cognitive performance compared to either aerobic PE or cognitive training alone, or to interventions involving other strategies; (2) to assess the quality of the studies performed to date; and (3) to make recommendations for future studies.

## 2. Methods

### 2.1. Search Criteria

We conducted a systematic literature search using the electronic databases PubMed and Web of Science (principal collection) for human studies published in English or Spanish in peer-reviewed journals from the earliest available record up to January 2021. The keywords of the PubMed search were combined with the following terms: (exercise OR physical activity OR fitness OR exergaming) AND (cognitive training OR cognitive rehabilitation OR neuropsychological rehabilitation) AND (cognition OR memory OR executive function OR attention OR neuropsychological tests) AND (stroke OR cerebrovascular accident OR brain ischemia OR poststroke). The following terms were combined for the Web of Science search: (stroke OR cerebrovascular) AND ((cognit * AND exer *) OR (cognit * AND physical)). Google Scholar was also searched (using the term “Combined exercise and cognitive training in Stroke”) for papers not indexed in PubMed and/or the Web of Science and gray literature. In addition, the reference lists from the articles chosen, as well as those from selected reviews, were manually examined to identify other potentially relevant manuscripts.

### 2.2. Study Selection

Studies were selected for inclusion according to the following criteria: the studies should be randomized controlled trials that (1) recruited stroke survivors ≥18 years old (ischemic or hemorrhagic stroke, any time since injury); (2) included an experimental condition comprising aerobic PE plus cognitive training, with or without additional elements; (3) included at least one validated neuropsychological cognitive test or battery with data reported at least at baseline and post-intervention; (4) the aerobic PE intervention should be supervised; (5) the parameters of the aerobic PE administered (intensity, duration, frequency, etc.) and the characteristics of the cognitive intervention should be specified; and (6) the cognitive training should be structured and target specific cognitive functions.

### 2.3. Data Collection and Extraction

Two members of the research team (E.R.-Q. and S.S.-S.) performed the initial search, removed titles and abstracts that were clearly beyond the scope of the review, and obtained the full text for all abstracts that either did not provide enough data for exclusion or which appeared to be potentially eligible for inclusion. All four authors then independently assessed the full text of the articles and selected the studies for inclusion based on the information from the full text.

Data were extracted from the full texts by two members of the review team (E.R.-Q. and S.S.-S.) using a standard template and was independently verified by the other two members of the research team (L.A.-A. and M.C.-A.). The extracted data included study, participant and intervention characteristics, and cognitive outcome data. To achieve a high standard of reporting, we followed the “Preferred Reporting Items for Systematic Reviews and Meta-Analyses” (PRISMA) guidelines [28,29].

**Figure 1:**
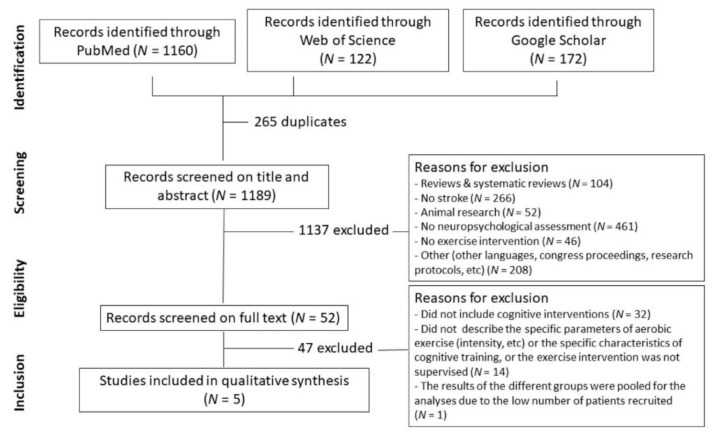
Flow diagram of literature search and selection of publications.

The results of the search and study selection process are shown in Figure 1.

### 2.4. Quality Assessment

The methodological quality of each of the randomized controlled trials included was evaluated using the revised Cochrane risk-of-bias tool 2 (Rob 2), according to the instructions [30]. Rob 2 includes five domains with potential bias: (1) the randomization process; (2) deviations from the intended interventions; (3) missing outcome data; (4) measurement of the outcome; and (5) selection of the reported result. The assessment quality was performed in duplicate by two different authors (M.C.A. and L.A.A.), and any discrepancies were discussed before a final joint decision was made.

## 3. Results

### 3.1. Study Characteristics

Five randomized controlled trials met the inclusion criteria. A sixth study was found that was initially designed as a randomized controlled trial [31]. However, in this study, and due to the low number of participants, the results of the three initial groups (combined aerobic PE + cognitive training, aerobic PE alone, and control) were pooled for a pre-post analysis, preventing us from carrying out a comparison between the different intended interventions.

The characteristics of the sample, the inclusion/exclusion criteria and the duration of the interventions of the revised studies are outlined in Table 1. Table 2 describes the experimental conditions, the characteristics of the aerobic PE and cognitive interventions, the outcome measures, and the main results reported.

### 3.2. Quality Assessment

As indicated in Table 3, the five randomized controlled trials were rated as having low risk of bias in most domains. In one case [33], the study was mainly aimed at detecting adherence to the intervention and was categorized as such in the Rob2 template. This study threw up some concerns with regard to the baseline characteristics (differences in the proportion of males and females; incidence of diabetes mellitus between the two groups), the fact that the assessment of the outcome variables was not blinded, and the significantly lower level of adherence in the control group compared to the combined intervention group. However, the authors took measures to reduce the impact of these drawbacks. Thus, the training and outcome assessments for a particular participant were performed by a different study member, to reduce assessment bias, and the cognitive outcomes were adjusted according to baseline characteristics.

### 3.3. Participants

The final sample sizes of the selected randomized controlled trials ranged from 30 to 179 individuals, with group sizes ranging from 12 to 83 participants. The sample sizes were based on power analyses in four studies [32,33,35,36], while the other work [34] indicated that the study was underpowered to detect clinical differences.

The mean age of the participants ranged from 50.63 to 64.59 years. The mean time elapsed since injury was less than 6 months in [32], less than 1 year in [33], and six months or more in the other studies [34,35,36].

The proportion of ischemic/hemorrhagic patients was only specified in three studies [33,34,35], but none of them examined whether the type of stroke had an influence on the cognitive outcome.

In all the studies, the participants had either mild cognitive impairment, as indicated by their Mini Mental State Examination (MMSE) or Montreal Cognitive Assessment (MoCA) scores, deficits in one or more neuropsychological tests (1.5 standard deviations below age and education-corrected norms), or subjective cognitive complaints. In contrast, the patients were not suffering from dementia.

Three studies [32,35,36] did not report the appearance of adverse events associated with aerobic PE. One study [33] reported adverse events, but these were all minor. Another study [34] reported the occurrence of training discontinuation in five participants, but this was due to comorbidities unrelated to aerobic PE.

Two of the studies specified the proportion of patients suffering hypertension and other cardiovascular disorders [33,35], diabetes/metabolic syndrome [35], or dyslipemia [33], and only one of them indicated the medications given to the patients [35]. Body mass index and years of education were specified in three studies [32,33,35], while fitness levels were only specified in one study [35]. The latter was the only one that examined the relationship between basal cognitive status (fluid intelligence), and basal fitness levels (as well as serum levels of IGF1 and BDNF) on post-intervention cognitive improvement. Both fitness levels and serum IGF-1 levels showed significant positive correlations with cognitive gains, but only the latter was significant when a regression stepwise analysis was performed.

### 3.4. Aerobic Exercise Intervention

The aerobic PE training involved a treadmill or bicycle ergometers [33,34,35,36], or jogging and cycling [32]. In two of the studies, the combined intervention also included either resistance exercise [33], or strength and balance training [32]. Exercise intensity was reported either as percentage maximum heart rate [33,34,36], percentage peak oxygen uptake [35], or ratings of perceived exertion (Borg’s scale) [32]. The exercise intensities used can be classified as moderate to vigorous.

### 3.5. Cognitive Training

Cognitive training consisted of computer-based training [32,33,35,36], or oral cognitive exercises carried out while walking on a treadmill [34].

### 3.6. Other Experimental Conditions

In the study by Bo et al. [32], three different experimental groups, in addition to the one combining aerobic PE and cognitive training, were included (exercise alone, cognitive training alone, and usual care plus video documentaries). Similarly, Ploughman et al. [35] also included three other conditions, but these comprised physical activity (range of motion, massage, and mobility tasks) plus cognitive training, physical activity plus games, and aerobic PE plus games. In the studies that only included two groups, the control interventions consisted of nonaerobic exercise (flexibility, muscle strengthening, and balance exercises) plus unstructured mental activities [36], a sham intervention (gentle stretching and range of motion exercise plus unstructured computer activities) [33], or aerobic training alone [34].

### 3.7. Duration of the Combined Interventions

The duration of the interventions was relatively short in all the studies: 10 weeks [34,35]; 12 weeks [32,33]; and between 12 and 18 weeks (depending on the number of weekly sessions) [36].

### 3.8. Cognitive Outcomes

As shown in Table 2, the cognitive outcome measures varied greatly between the studies, and included the trail making test, part B (TMTB) [33], Stroop tests [32,33], digit span [32,33], mental rotation [32], fluid intelligence (RAVEN’s progressive matrices test) [35], several tests from the Wechsler Adult Intelligence scale (WAIS), including memory scale, spatial span, and verbal paired associates [36], Montreal Cognitive Assessment (MoCA) scores [33,34], and dual motor-cognitive tasks [34]. In addition, one of the studies [33] included several other tests, such as Hopkins verbal learning test, delayed recall, grooved pegboard, Delis-Kaplan executive function test, digit symbol-coding, substitution test, brief visuospatial memory test, and the CogState brief battery test.

### 3.9. Post-Intervention Effects

Within-subject analyses indicated that several interventions were associated with improved performance in the post-intervention period, or follow-up assessment compared to baseline. Improvements were found only after the combined aerobic PE + cognitive training condition in Stroop tests and mental rotation [32], RAVEN’s progressive matrices test [35], MoCA scores [33,36], spatial span [36], and cognitive responses during dual task-walking [34]. In addition, aerobic PE alone was associated with pre-post improvement in TMTB and forwards digit span [32].

Only two of the studies compared the combined aerobic PE + cognitive training intervention with either aerobic PE alone [33,34] or with cognitive training alone [32]. The combined intervention led to better performance than aerobic PE alone, in terms of digit span and mental rotation, and in mental rotation compared to cognitive training alone [32]. In contrast, no significant differences in MoCA scores and cognitive responses during dual-task walking were found between aerobic PE + cognitive demand and aerobic PE alone [34]. In the other studies, the combined aerobic PE + cognitive training condition was compared to other kinds of combined interventions, such as physical activity plus cognitive training, physical activity + games, or aerobic PE + games [35], or to combinations of unstructured cognitive and motor training tasks [33,36]. The combined aerobic PE + cognitive training condition led to greater improvement compared to motor training + unstructured mental activities in both MoCA scores and spatial span [36], and in MoCA scores when compared to the sham condition (supervised training involving gentle stretching and range-of-motion exercises, as well as computer games and word searches), but the statistical significance was lost when the data were adjusted according to the baseline characteristics [33]. Compared to the usual care administered (with neither aerobic PE nor cognitive training), the combined aerobic PE + cognitive training intervention was associated with higher scores in the TMTB (divided attention), a Stroop task (inhibitory capacity), digit span (working memory), and mental rotation [32]. In contrast, Ploughman et al. [35] found no significant differences between the different interventions with regard to the pre-post improvement in RAVEN’s progressive matrices tests (although they did in the follow-up assessment).

Meta-analyses were not performed due to the variability in the specific cognitive outcome measures and experimental conditions, and to the low number of studies that met the selection criteria.

### 3.10. Follow-Up Outcome

A very interesting question is whether any benefits of the combined interventions are maintained for a time once they have been discontinued. However, follow-up cognitive assessment was only reported in three of the studies. In these studies, the follow-up assessment took place at either 3 [34,35] or 6 months [32] post-intervention. The latter study did find that the cognitive effects of the combined intervention were maintained at the 6-month follow-up for all the cognitive tests that had improved at the post-intervention assessment. The aerobic PE + cognitive training condition was associated with greater improvement in fluid intelligence at the follow-up assessment compared to the baseline values (while the pre-post comparison showed no significant differences between the groups) [35]. When comparing the follow-up and baseline data, no significantly higher cognitive gain after the combined intervention was found for the cognitive responses during dual-task walking [34]. On the contrary, there was a small difference in favor of the group who did not participate in the combined intervention.

## 4. Discussion

Interventions that combine the use of aerobic PE with cognitive training have yielded positive results in terms of reducing the cognitive disruption associated with aging and mild cognitive impairment [25,26]. Cognitive rehabilitation strategies and aerobic PE, applied alone, can also benefit cognitive impairment associated with stroke. One could assume that the effectiveness of these kinds of interventions would be enhanced when combined. Despite this, the results of the selection process clearly revealed the scarcity of controlled studies assessing the influence of combined supervised aerobic PE + cognitive training interventions on cognitive function in stroke patients. Interestingly, the five randomized controlled trials analyzed here were published in 2019 and 2020, indicating the growing interest in this topic. Only two of these studies compared the effects of aerobic PE + cognitive training with those of either aerobic PE alone [32,34] or cognitive training alone [32]. The remaining three studies used other kinds of combined interventions as their control conditions [33,35,36]. These “sham” combined conditions may demand higher mental and physical resources than single interventions, meaning it may be more challenging to demonstrate whether aerobic PE + cognitive training is significantly superior when the comparison groups involve more than one intervention, even if these comprise unstructured activities. In this regard, several studies have shown the cognitive benefits of multimodal interventions (which usually include physical activity or PE and cognitive training, in addition to encouraging other healthy lifestyle habits) in stroke patients (for example, [38,39]).

In one of the studies analyzed here [33], the combined intervention included resistance training in addition to aerobic PE and cognitive training. There is evidence that combining aerobic and resistance exercise maximizes the positive effects of PE in healthy aging [40]. Similarly, the results of a meta-analysis indicated that, in stroke patients, different kinds of physical activity, such as balance, resistance, and muscular strength training, confer health benefits that are complementary to those of aerobic PE [41]. The results of several randomized controlled trials show, in fact, that combinations of PE with other kinds of physical activity or motor training exercises have also demonstrated cognitive benefits in stroke patients [24,42,43,44,45,46]. Similarly, combining nonaerobic PE with cognitive tasks can also induce cognitive benefits. For example, low-intensity golf training (mainly involving coordination exercises) combined with cognitive tasks led to greater improvement in mental rotation (but not attention and visual spatial memory) than a control condition consisting of social communication meetings [47]. Additionally, a combined intervention comprising aerobic PE, resistance, and balance motor training in addition to recreation and leisure activities requiring planning, strategy, decision, memory and learning (playing billiards, bowling, arts and crafts, and cooking) improved the performance of stroke patients in Stroop tasks, forwards and backwards digit span, and motor function (6 m walking test), compared to a waiting list control group [48].

Another way to deliver a combination of exercise and cognitive training is through exergames. We included the term “exergames” in our literature search, but we did not include the resulting studies in our final selection as the specific characteristics (particularly, intensity) of the PE intervention were not reported. Nonetheless, exergame interventions have also been associated with improved cognitive function. For example, a study [49] reported that, compared to standard care (involving walking, physiotherapy, speech therapy, and neuropsychological rehabilitation), Nintendo Wii Sports resort games plus usual care led to higher scores in the trail making test, part A, TMTB, forwards digit span, and total digit span, although these differences were not significant when the scores were normalized according to the percentiles of the general population (the effect sizes were nonetheless still higher in the exergames + standard care group).

A controversial question is whether the cognitive effects of aerobic PE are higher than those of balance, stretching, or resistance exercise. One study [50] found no significant differences in the degree of improvement of forwards digit span between patients submitted to aerobic PE compared to those that received a balance and flexibility intervention. In contrast, aerobic PE led to a greater improvement in Stroop tasks compared to balance and flexibility exercise, but only in females [51], which raises the interesting question of gender differences in the cognitive effects of either or both kinds of exercise modes. However, in a systematic review, these authors found no clear evidence of any gender differences based on existing studies (it must be noted though that these studies were not aimed at testing gender differences) [52].

The low number of studies combining aerobic PE + cognitive training does not allow us to ascertain whether certain cognitive components are more favored than others using this combination. The revised studies suggest that pre-post improvements can be found after both aerobic PE + cognitive training and aerobic PE alone in processing speed and divided attention tests (TMTB) [32], while the combined intervention was superior to aerobic PE alone and to other control conditions with regard to spatial and non-spatial working memory (forwards digit span and spatial span) [32,36], visuospatial function (mental rotation) [32], fluid intelligence [35], and MoCA scores (a scale including several cognitive components) [33,36]. In healthy aging subjects, aerobic PE seems to be particularly effective at improving executive function and processing speed [40]. Processing speed and attention (but not executive function) were the main cognitive domains that resulted in significant improvements after physical activity training, according to a meta-analysis of studies on stroke patients [18]. However, the effects of interventions that also include cognitive training, in addition to PE, may also depend on the specific cognitive subdomains primarily targeted by the cognitive rehabilitation exercises.

Multiple variables may influence the cognitive effects of interventions after stroke, such as time since injury, duration of the intervention, specific exercise characteristics (frequency, intensity, etc.), and so on. The revised studies differed particularly in regard to time from injury. Two of the studies recruited patients at the subacute stage (less than 6 months [32] or one year post-stroke [33]), while the other works included patients that had suffered stroke more than 6 months prior (in the majority this was several years beforehand). It is expected that the influence of interventions that target plasticity mechanisms, such as exercise and cognitive rehabilitation, may differ depending on whether the brain is still being affected by the spontaneous recovery mechanisms that are maximal during the first weeks and months post-injury, or after this “temporal window of opportunity” has closed [20]. Thus, on the one hand, it seems advisable to initiate these kinds of interventions soon after a stroke has been suffered, yet, on the other hand, the benefits of early interventions may be masked, at least in the short term, by the spontaneous recovery mechanisms also experienced by patients submitted to standard care, which is usually much more intense at the subacute stage compared to the chronic stage. Animal research suggests that early (but not immediate) exercise initiation is associated with greater neuroprotection and plasticity effects and, subsequently, to better cognitive outcomes than late-onset exercise [53,54]. On the other hand, early initiation must be done with caution in patients, given that starting physical activity under conditions of altered cerebral blood flow or uncontrolled blood pressure may be harmful [55]. In this review, the benefits of combining aerobic PE with cognitive training have been reported both for subacute and chronic patients. This is in line with the studies using aerobic PE or aerobic PE + motor training or other interventions. In a systematic review and meta-analysis, benefits of aerobic PE alone or aerobic PE combined with nonaerobic PE interventions were found to exert benefits not only when initiated early after injury, but also in chronic stroke patients [18]. Overall, it seems that either the “window of opportunity” of maximal spontaneous plasticity is longer than expected or that exercise may also target neural mechanisms that are still at play long after injury.

The duration of the intervention is another factor that may influence the benefits observed. However, no conclusion can be drawn based on the studies analyzed here, since they all used very similar intervention durations, ranging from 10 to 12 weeks. It does seem that interventions with physical exercise lasting 12 weeks are enough to produce cognitive benefits [18], although whether these benefits are long lasting or not is less well understood.

The optimal exercise intensity for cognitive improvement after brain damage is far from clear [53]. Animal research suggests that moderate intensities are better than high intensities at promoting recovery, probably because the latter may exacerbate stress responses [16,53,54]. However, high-intensity interval training, which includes repeated short bouts of high-intensity exercise, has been successful at increasing plasticity markers in animal stroke models [56,57], suggesting that it could also exert cognitive benefits. Moreover, some of the molecular effects of exercise are intensity-dependent. For example, a bout of exercise on a treadmill produced intensity-dependent increases in brain-derived neurotrophic factor (BDNF) and vascular endothelial growth factor (VEGF) in serum, and, to a lesser extent, insulin-like growth factor 1 (IGF-1), in stroke patients [58]. The exercise intensities used in the revised studies were not very different and can be all be classified as moderate or moderate-to-vigorous. In addition, fitness levels were only recorded in one study [35], and only the aerobic PE + games group showed a significant pre-post improvement in cardiovascular fitness (VO2peak), while the increase found in the aerobic PE + cognitive training group was not significant. Interestingly, a significant positive correlation was found between the baseline VO2peak and cognitive improvement at follow-up. The same study was the only one that also analyzed molecular biomarkers (serum levels of BDNF and IGF-1). They found no differences between the groups in the levels of either of these neurotrophins, but there was a significant positive correlation between IGF-1 upregulation after an acute bout of exercise and the degree of cognitive improvement. Other studies have also demonstrated relationships between several biomarkers and cognitive improvement. For example, a treatment combining aerobic PE + physiotherapy has been reported to increase serum BDNF levels (compared to physiotherapy alone), and a positive correlation was found between BDNF upregulation and the patients’ cognitive performance [59]. This combined intervention was also associated with increased flow in the middle cerebral artery.

Besides the cognitive effects, four of the revised studies [33,34,35,36] also analyzed other outcome measures, such as quality of life, mood, and motor function. One study [36] reported that improved motor function (6 min walking test) in the aerobic PE + cognitive training condition was greater than in the control intervention, while another work [34] found that the combined intervention was associated with higher increases in self-reported physical activity than for the control condition, but linked to similar increases in walking distance and confidence in community walking in both groups. Quality of life was similarly improved in the aerobic PE + cognitive training and control groups [33,34], or not improved in any of the intervention conditions [36]. Finally, improved mood was reported after both the combined intervention and the sham condition [33], but another study [35] failed to demonstrate a significant improvement in depression scores for any group.

Limitations. The main limitation of this work is the low number of studies that fulfilled the inclusion criteria, which, in addition to the variability in the specific cognitive assessment tools and in the experimental conditions used, makes it hard to reach clear-cut conclusions. Under these circumstances, quantitative synthesis and meta-analyses were deemed not very informative and were, thus, not performed.

Another limitation is that only three studies examined the cognitive outcome after a follow-up period.

In addition, none of the studies examined whether the effects of the combined intervention varied depending on several variables that are known to have an influence on the cognitive status of stroke patients, such as cardiovascular disorders, hypertension, diabetes, body mass index, medications, and years of education [60,61].

Finally, none of the studies examined, either, the possibility that the effects of the interventions might vary depending on the type of stroke (mainly ischemic versus hemorrhagic).

## 5. Conclusions

Are interventions that combine aerobic PE and cognitive training more effective to reduce the cognitive deficits of stroke patients compared to each intervention alone or to other combined interventions? More controlled studies assessing the joint and separate influence of exercise and cognitive training on cognitive function in stroke patients are required to answer to this question.

Several strategies can be recommended for future studies. First, given the high inter-individual variability of the specific cognitive deficits that each patient may present, it would seem advisable to maximize the use of statistical methods that allow the baseline characteristics of each patient to be controlled (age, ischemic/hemorrhagic stroke, years of education, cardiovascular and metabolic disorders, etc.). Even more, these baseline characteristics could be used to deliver statistical predictive models, although this kind of studies requires the recruitment of large numbers of patients per group, which is particularly challenging. The addition of biochemical measures, neuroimaging analyses, and fitness-related measures would shed light on the specific neural mechanisms involved in the different intervention parameters (duration, frequency, intensity, kind of cognitive function predominantly targeted by cognitive training, etc.). Finally, the inclusion of follow-up assessments would help determine if the influence of the intervention is short-lived and fades once the intervention is discontinued, or the benefits are maintained in the long term.

## Figures and Tables

**Table 1:** Patients’ characteristics, inclusion/exclusion criteria, and duration of the interventions of the studies included in the systematic review.

Reference	Sample Size	Age	Time since Injury	% Ischemic/Hemorrhagic Stroke	Inclusion/Exclusion Criteria	Duration of Intervention
[32]	Initial sample: 225 Final sample: 179	64.59 ± 4.27 years	<6 months	NS	Inclusion criteria: aged over 18 years; medically stable; able to understand and follow verbal instructions; meet the diagnostic criteria for vascular cognitive impairment. Exclusion criteria: severe somatic diseases or mental disorders, including anxiety and depression; visual or auditory disturbances in recent months; motor impairment.	12 weeks
[33]	Initial sample: 131 Final sample: 94 (69 in combined intervention group; 25 in sham intervention group)	59 ± 11 years in combined group and 58 ± 12 years in sham intervention group	<1 year	% ischemic: 81% in combined intervention group; 84% in control group	Inclusion criteria: age >18 years; modified ranking scale ≤3; less than 75 min/week of vigorous physical activity or less than 150 min of moderate physical activity. Exclusion criteria: neurodegenerative diseases or unstable medical and psychiatric conditions.	12 weeks
[34]	Initial sample: 50 (26 and 24 participants per group) Final sample: 45 (24 in aerobic PE with simultaneous cognitive training group; 21 in PE alone group)	60.85 ± 14.38 in aerobic PE with simultaneous cognitive training group; 62.25 ± 15.53 in PE alone group	≥6 months	Ischemic:18/26 and 13/24 patients Hemorrhagic: 7/26 and 10/24 patients Both: 1/26 and 1/24 patients	Inclusion criteria: ≥18 years; reduced 2-min walk distance or a visibly abnormal gait; able to walk on a treadmill. Exclusion criteria: concurrent neurological conditions or psychological disorders; contra-indications to exercise.	10 weeks
[35]	Initial sample: 60 Final sample: 52	63.4 ± 11.3 years	>6 months (mean time since stroke: 3.4 years)	% ischemic: 77% (9/12; 12/13; 11/15 and 8/12)	Inclusion criteria: ischemic or hemorrhagic stroke; self-reported cognitive problems related to stroke interfering with daily functioning; ability to perform 2-step instructions; ambulation with/without aid ≥10 m; negative high-risk screening; agreement to refrain from AE outside of trial intervention.	10 weeks
[36] (See [37] for more details)	30 (*N* = 15 per group)	50.63 ± 3.99 in sequential PE + cognitive training group and 60.21 ± 3.10 in control group	≥6 months	NS	Inclusion criteria: Ischemic or hemorrhagic stroke; ≥19 in MMSE; <26 in MoCA; self-reported or informant-reported memory or cognitive complaints or score on the Clinical Dementia Rating scale ≤0.5; able to follow the study instruction; adequate cardiopulmonary function to perform aerobic PE; able to walk with or without assistive devices. The participants were stratified according to MMSE scores (strata 1: MMSE score: 19–24; strata 2: MMSE score: 25–30).	Between 10 and 18 weeks, depending on number of weekly sessions

**Abbreviatures:** MMSE: Mini Mental State Examination; MoCA: Montreal Cognitive Assessment; NS: not specified; PE: physical exercise.

**Table 2:** Experimental conditions, characteristics of the interventions, and main outcomes obtained in the revised studies.

Reference	Experimental Conditions	Characteristics of the Aerobic PE Intervention	Characteristics of Cognitive Training	Measures of Cognitive Assessment	Follow-Up	Other Outcome Measures	Main Outcomes
[33]	Focus group: Combined aerobic PE + resistance exercise + cognitive training Control group: Sham intervention	Each session consisted of combined aerobic PE + resistance exercise (40–60 min) + cognitive training (40 min) (only aerobic PE in 2 of the 3 weekly sessions). Aerobic PE was done in treadmill or bicycle ergometer. Target intensity: from 50 to 65% of MHR	Using an adaptive computerized platform from Brain Fitness Program. Each session consisted of four 10-min training tasks targeting attention, memory, psychomotor speed, and working memory	General cognitive screening (MoCA) Verbal learning and memory (recognition discrimination and recall): HVLT Manipulative dexterity and fine motor speed: grooved pegboard inhibitory capacity; Stroop; CogState brief battery Comprehensive verbal and not verbal executive function assessment (inhibitory capacity, switching, color naming, etc.): DKEFS Processing speed, working memory, visuospatial processing, and attention: CogState brief battery Visuospatial memory: BVMTR Working memory: BDS	No	Mood (center of epidemiological Studies—Depression Scale) and quality of life (SIS). Physical strength and mobility Measures of feasibility, safety, and adherence (main outcome of the study)	The analyses of cognitive function were done by grouping those tests assessing the same cognitive domain. Pre-post improvement: MoCA scores: only in the combined intervention Mood and SIS: improved similarly in both groups Between-group differences The combined intervention led to better MoCA scores than the sham intervention, but the between-group differences were not significant when adjusted by baseline characteristics.
[32]	Focus group: Combined aerobic PE + cognitive training Other experimental groups: Aerobic PE alone Cognitive training aloneControl (usual care + video documentaries)	Each exercise session lasted 50 min and consisted in a warm-up period (5–10 min) of aerobic PE (jogging or cycling); 30–35 min of endurance exercise (aerobic PE), strength, and balance; and a cool-down period (5 min) of stretching exercises. Target intensity: Borg’s scale: 13–15 (somewhat hard; moderate)	Computerized cognitive training (COGPACK program) carried out in group and under supervision. It included 64 programs in areas of visual motor learning, memory, attention, and executive function.	Divided attention, processing speed, and cognitive flexibility (TMTB) Inhibitory capacity (Stroop) Working memory (FDS) Visuoperceptive function (mental rotation)	Yes: 6 months	NS	Within group pre-post improvement: TMTB: in combined intervention, cognitive training alone and aerobic PE alone Stroop: only in combined intervention FDS: in combined intervention and cognitive training alone Mental rotation: only in combined intervention Between group comparison at post-intervention: TMTB: combined intervention) > control Stroop: combined intervention) > control FDS: combined intervention > control; combined intervention > aerobic PE alone; cognitive training alone > control Mental rotation: combined intervention > control; combined intervention > aerobic PE alone; combined intervention > cognitive training alone Follow-up: The cognitive gains were only maintained in the combined aerobic PE + cognitive training group.
[34]	Focus group: Aerobic PE in a treadmill + cognitive demand Control group: Aerobic PE without simultaneous cognitive demand	30 min/session Target intensity: 55–85% MHR	Cognitive tasks were carried out while exercising. The tasks were given orally or visually, and included auditory Stroop, serial subtraction, clock-face task, letter fluency, alternative uses, creativity, listening tasks, and planning of activities of daily living	General cognitive screening (MoCA) Dual task effect on walking and cognition (performance on cognitive task when walking, and on walking when doing a cognitive task as compared with doing task alone)	Yes: 3 months	Barthel ADL Physical activity (StepWatch activity monitor and PASE), SF-36 and EuroQol-5D	Within-group pre-post improvement: In both groups: significant increase in cognitive response during dual task-walking. Both groups also showed increased walking distance, confidence in community walking, and quality of life (total SF-36 score and EQ-5D index). Between-group comparisons: Change from baseline to follow-up: The aerobic PE + cognitive demand group showed a significantly higher increase in PASE scores compared to the aerobic PE without cognitive demand group. A small difference, in favor of the group without simultaneous cognitive demand, was found for the increase in cognitive responses during dual task walking.
[35]	Focus group: Aerobic PE + cognitive training Other groups: Physical activity (range of motion, massage) + cognitive training Physical activity + gamesAerobic PE + games	50–70 min per session Target intensity: 60–80% of VO2peak	Cognitive training: Computerized dual-n-back training. Level of difficulty adapted to the individual’s performance Games: participants sat at a workstation and played a non-adaptive computer-based game that involved strategically placing descending puzzle pieces.	Fluid intelligence (RAVEN’s progressive matrices test)	Yes: 3 months	HADS-D Aerobic fitness (VO2peak) Serum levels of BDND and IGF-1	Within-group pre-post improvement: Combined Aerobic PE + cognitive training showed the highest improvement in fluid intelligence. Between-group differences in raw change from baseline to follow-up: Fluid intelligence: Combined aerobic PE + cognitive training > aerobic PE + games. However, these differences were lost with Bonferroni corrections. No significant differences between groups in IGF-1 levels, but patients with higher upregulation of IGF-1 serum levels after an acute bout of exercise showed the higher cognitive improvement at follow-up. A significant positive correlation was also found between baseline VO2peak and cognitive improvement at follow-up.
[36] (see [37] for more details)	Focus group: Sequential aerobic PE in a stationary bicycle + computerized cognitive training (30 min) Control group: Nonaerobic PE (30 min; muscle strength, flexibility, balance) + unstructured mental activities (30 min)	60 min per session 2–3 sessions/week Target intensity: 40–70% MHR	Computerized cognitive training using BrainHQ program, which was used to train attention, recognition, color and shape identification, calculation, visual perception, visuospatial processing, and executive function.	General cognitive screening (MoCA) Explicit verbal memory (WAIS verbal paired associates) Spatial working memory (WAIS memory scale)	No	6MWT IPAQ EuroQoL-5D Community Integration Questionnaire	Within-group pre-post improvement: MoCA, spatial working memory, and 6MWT: only in sequential Aerobic PE + cognitive training No significant improvement, in any group, in transfer of sequential training to social participation and quality of life Between group differences at post-intervention assessment: MoCA, spatial working memory, and 6MWT: sequential aerobic PE + cognitive training > control

**Abbreviatures:** ADL: activities of daily living; BDNF: brain-derived neurotrophic factor; BDS: backwards digit span; BVMTR: Brief Visuospatial Memory Test; Euro-Qol-5D: European Quality of Life, 5 dimensions; DKEFS: Delis–Kaplan Executive Function test: FDS: forwards digit span; HADS-D: Hospital Anxiety and Depression Scale (Depression Subscale); HRR: heart rate reserve ; HVLT: Hopkins Verbal Learning Test, Revised; IGF-1: insulin-like growth factor 1; IPAQ: International Physical Activity Questionnaire; MHR: maximum heart rate; MoCA: Montreal Cognitive Assessment; NS: not specified; PASE: Physical Activity Scale for Elderly; PE: physical exercise; SF-36: The 36-Item Short Form Health Survey; SIS: Stroke Impact Scale; TMTB: trail making test, part B; VO2peak: maximum oxygen consumption; WAIS: Weschler Adult Intelligence Scale; 6MWT: 6 Minute Walking Test.

**Table 3:** Risk of bias of the five randomized controlled trials that were selected.

Reference	Randomization Process	Deviations from Intended Interventions	Missing Outcome Data	Measurement of the Outcome	Selection of the Reported Result	Overall
[32]	+	+	+	+	+	+
[33]	+	¡	+	+	+	¡
[34]	+	+	+	+	+	+
[35]	+	+	+	+	+	+
[36]	+	+	+	+	+	+

Legends: +: low bias ¡: some concerns.
